# Caring for the lonely: an ethical case for social prescribing against loneliness

**DOI:** 10.1007/s11019-026-10386-8

**Published:** 2026-08-21

**Authors:** Zohar Lederman

**Affiliations:** 1 Department of Emergency Medicine, LKS Faculty of Medicine, Hong Kong University, Hong Kong, Hong Kong; 2 University of Hong Kong, Centre for Medical Ethics and Law, Hong Kong, Hong Kong

**Keywords:** Loneliness, Social prescribing, Justice, Cost-effectiveness

## Abstract

Social prescribing to mitigate loneliness has been gaining traction worldwide in the past two decades. Patients found to be lonely by their clinicians are linked with social services and are encouraged to engage in non-clinical, social activities such as painting and music classes, Tai Chi, outdoor excursions etc. The science grounding the cost-effectiveness of these interventions, however, has been scarce. From a justice perspective which prioritizes resource allocation based on desired outcomes, social prescribing may be hard to justify. Care ethics, however, may be more adept at justifying and indeed compelling the investment of limited public resources in social prescription activities aimed at reducing loneliness. The assumption is that individuals have an entitlement to such activities first because they have a right to health, and second because, as inherently social creatures, they have a right to not be lonely.

## Introduction

A young patient comes to the emergency department complaining of pain throughout her body for the last two months. Barring bare necessities, she had not left her home for 6 months. Having immigrated from another country without her family, she feels lonely. She had never been evaluated for a major depressive disorder. The only care I could offer was connecting her with a social worker.

Loneliness is a major public health issue, negatively affecting people’s health and wellbeing (Lederman 2023a, b, c, Commission on Social Connection 2025) The focus here is on the most traditional understanding of loneliness, according to which loneliness is a distressing mismatch between one’s perceived social interactions and one’s expectations thereof. (Commission on Social Connection 2025) I acknowledge yet set aside the idea that other kinds of loneliness exist, and that some may be more detrimental to human health and flourishing than others (Lederman 2023a, b, c). Loneliness correlates with but conceptually differs from social isolation which is the objective, physical phenomenon of aloneness. Because of its ubiquity and serious adverse health effects (Hawkley 2022), a Commission on Social Connection was recently established by the World Health Organization (WHO) with the mandate to address loneliness as a,

“pressing health threat, promote social connection as a priority and accelerate the scaling up of solutions in countries of all incomes.”(2023)

As seen for instance in debates surrounding the national distribution of vaccines against Covid-19, deciding the criteria for a national distribution of resources for such a purpose will undoubtedly be challenging: should they be distributed based on need, meaning for instance high prevalence of loneliness in a specific geographical area or group, or rather based on potential effectiveness or cost-effectiveness in a specific geographical location or group?

Either way, a just distribution would depend on our normative evaluation of what makes loneliness a bad thing: is it only because it negatively affects health, or is it because being lonely undermines our wellbeing and our very essence as social beings? (Lederman 2023a, b, c; Lederman 2026). In the former case, resources might preferably be allocated to those who are socially isolated rather than lonely, as the evidentiary link between social isolation and ill-health is stronger than that between loneliness and ill health (2020). In the latter case, preference may be given to those most at risk for loneliness, e.g. individuals aged 18–24 (Barreto et al. 2021, Switsers et al. 2022). This may seem as counter-productive from a cost effectiveness analysis aimed at reducing premature mortality, because this age group is overall less likely to suffer from the chronic diseases that are associated with loneliness, e.g. cardiovascular disease and stroke. Similarly, certain forms of loneliness, such as that affecting the patient mentioned above, may empirically turn out to be more detrimental for health than other forms- should they be prioritized?

Epistemic and transitional justice may be coupled with distributive justice to drive resource allocation somewhat differently. A recent paper links the focus in the loneliness literature on individuals to neoliberalism and the Western ideals of autonomy and self-reliance, calling for a structural social justice approach (Barreto et al. 2024). A recent book suggests that neoliberalism that prioritizes economic and industrial development above all has led to the atomization and estrangement of individuals from their communities, community values, and from other individuals in their community. This has eroded civic values and the feeling of being connected to one’s community, leading to political loneliness. This kind of loneliness manifests in people’s felt disconnect from and distrust in their liberal governments on the one hand, and increased tribalism and the lore of authoritarian regimes on the other. Solutions to this kind of loneliness should then not focus on individuals but rather on the structure of governments and communities, promoting public spaces that encourage wide public participation. The goal should be to return to civic republicanism as imagined by Hannah Arendt, where people speak and act with one another in the pursuit of creating a shared rather than an atomized world (Gaffney 2020; Lederman 2021).

While these accounts are right that a structural rather than an individualistic approach to loneliness mitigation is preferable in addressing the different kinds and mechanisms of loneliness (Lederman 2023a, b, c), a social justice approach would still require adjudicating between lonely individuals who deserve public support and those who do not, or those who need more compared to others. Bill Gates (2024), or the imaginary Bruce Wayne are well-off and yet may be lonely- do they not deserve public support in mitigating their loneliness? Similarly, some forms of mitigation may be preferable over others, particularly perhaps in the case of sexual or erotic loneliness (Lederman 2026).

Issues of distribution are bound to be highly complex; they will necessarily require inter-disciplinary and intercultural, multi-stakeholder deliberations. Questions would refer to the normative theoretical approach guiding such mobilization- should it be based on social justice? What specific account of social justice? Should it be based on other theoretical approaches in public health, e.g. solidarity?

This paper espouses a care ethics approach in arguing that resource allocation for mitigation efforts against loneliness should be based primarily on need. The ‘need’ here refers to the knowledge of being, and in fact being, part of a community that cares for its lonely members or those who are at risk of being lonely. The need is a need for care, not for specific resources, policies, or interventions. These matter and should be evaluated based on their effectiveness to mitigate loneliness and reduce its negative health and wellbeing impacts. But more importantly, they matter and should be evaluated based on their expression of care and whether they are perceived to express care by the lonely.

Care ethics has been espoused in the context of technology-mediated healthcare practices, where authors have explicitly prioritized healthcare policies based on care ethics rather than maximum efficiency (Ramvi et al. 2023). What care ethics means in the context of technologies used to mitigate loneliness, e.g. social robots and digital humans, deserves further attention.(Lederman 2023a, b, c; Lederman and Jecker 2023, Jecker et al. 2024) When it comes to social prescribing against loneliness, however, in light of the available evidence, a public health policy that is grounded in care ethics rather than justice seems more adept. It is more adept because the main question it asks is ‘what matters’, rather than ‘what works.’ And what matters is social connection with other humans: that human communities are actively engaging with the issue of loneliness while striving to maintain an ideal of human society (Brownlee 2020; Lederman 2021).

Social prescribing (SP) is one of the most common kinds of interventions against loneliness. It consists of non-clinical interventions aimed at reducing loneliness among community dwellers. SP usually involves assigning paid or unpaid link workers, otherwise known as health connectors, community connectors, community navigators, or village agents, to people who are lonely or socially isolated or who are at risk of being lonely. This is done through collaborations among clinicians, community-based organizations, social care services, and public health agencies (Liebmann et al. 2022) Specific activities that would then be facilitated by these workers include health-related support groups, community gardening, arts and recreational activities, exercise classes, luncheon clubs, and welfare rights advice (Liebmann et al. 2022) These activities may require substantial resources, both monetary and otherwise.

The main argument advanced here is that caring in the context of loneliness and SP means supporting SP programs even if their effectiveness is empirically uncertain. This care ethics approach contrasts with a justice approach according to which mitigation efforts should be based primarily on cost-effectiveness analyses.

The strategy of this paper is as follows. Section "Empirical evidence of social prescribing" reviews the empirical literature of SP, concluding that there is limited robust evidence to support it. Section "Care ethics and public policy" defines and critically reviews some of the literature on care ethics. Section "Loneliness and SP" asserts a right against loneliness and applies care ethics to loneliness and SP.

Before proceeding, however, two clarificatory remarks are in order. First, the paper focuses on one specific kind of interventions against loneliness-SP. Deliberations about other kinds of interventions, e.g. digital interventions or pet-assisted care (de Vries 2021), may conclude differently, based on the available evidence as to their effectiveness as well as our normative evaluation of them (Lederman 2023a, b, c; Lederman and Jecker 2023, Welch et al. 2023).

Second, the paper treats loneliness as a homogeneous experience across time, cultures, sexes, and ages. This is likely to be overly simplistic. As loneliness is necessarily subjective, it depends not only on one’s expectations of social relations but also on one’s perception of one’s existing social relations. Both of these, however, are socially construed to a large degree. One’s expectations of the quantity and kind of social relations depends on how one sees oneself, one’s perception of one’s social status, and how one perceives one’s community. These depend in turn on traits that are inherent to the individual, including age, race (loosely defined), sex, character etc. For example, being lonely negatively affects all of these, creating a vicious cycle where loneliness begets social isolation which in turn increases loneliness. One’s expectations, however, also depend on one’s societal norms and expectations, and these depends on the time period in which one lives, one’s country, one’s social circles etc. Women in a certain society, for instance, may be expected to be friendlier and have wider social circles than men, and this would in turn influence their judgment of their own social standing. Similarly, individuals in a society that is more communal compared with a society that is more individualistic often internalize the local ethos and set their social expectations accordingly. In other words, a woman in Asia and a man in the US may have completely different criteria to decide for themselves whether they are lonely, both in terms of expectations and in judging their existing social relations (Alberti 2019; Luhmann et al. 2023, WHO 2025) This paper sets aside this complexity as an academic exercise to clarify the main argument as much as possible, but if the argument is then to be found compelling, it should be implemented appropriately with this complexity in mind.

## Empirical evidence of social prescribing

Although generally favorable towards SP for health and social outcomes that go beyond loneliness, authors have lamented the lack of robust evidence to justify large investments in what they designate as systems, rather than interventions of SP. They particularly note three challenges in research pertaining to SP. First, SP is necessarily context-dependent and heterogenous in nature, so defining what SP is or what exactly it includes is not straightforward. The fact that SPs are systems with multiple components means that evaluating these components is also not straightforward. ‘Success’, then, becomes hard to define and therefore to quantify. Second, the fact that SP systems depend on local factors for their effectiveness and implementation means that they are hardly generalizable to other national and international contexts. Third, conducting research to evaluate SP systems is cumbersome, because of the need for instance to create local collaborations while maintaining the autonomy of the researchers, and to appropriately select control groups (Husk et al. 2019).

A 2017 systematic review of SP in general (meaning, not for loneliness mitigation) examined UK-based studies and reports published from 2000–2015. Outcomes included an increase in self-esteem and confidence, improvement in mental wellbeing and positive mood, and reductions in anxiety, depression and negative mood (Chatterjee et al. 2017).

SP activities included the following:

- ‘Arts on prescription’: programs that offered creative and participatory workshops of dance, drama, music, painting, and poetry.
- Books on prescription or Bibliotherapy: programs that allowed participants to borrow self-aid books.
- Education on Prescription: programs that enabled participants to engage in various learning activities.
- Exercise on Prescription: programs that encouraged participants to engage in social exercise activities.
- Ecotherapy: programs that encouraged patients to engage in outdoor activities.
- Healthy Living Initiatives: programs that targeted disadvantaged sectors of the population.
- Information Referral: programs that provided participants with general information pertaining to finances, welfare services etc.
- Programs that facilitated mental health services.
- Time banks: programs in which participants could volunteer specific amounts of time to assist others and receive the same assistance in return on another occasion.

The authors lament the lack of rigorous evaluation and statistical analysis in many of the reports, questioning their scientific contribution.

The evidence from SP that specifically target loneliness originates primarily from the UK and is indeed limited due to high heterogeneity in terms, methodology, and outcome (Reinhardt et al. 2021). Outcomes evaluated may be reduction in loneliness or social isolation, return to work, reduction in suicide rates or depression, increased wellbeing, and health utilization including recurrent visits to the clinic and hospitalizations. The question of who determines how the outcome is evaluated, or how success is to be defined, then, should always be kept in mind.

Perhaps the most known ‘success’ story of SP is the ‘Compassionate Frome Project’ that included all of the 28,000 inhabitants of the town of Frome in Somerset, UK. Born out of a collaboration between a community development worker named Jenny Hartnoll and a general practitioner named Helen Kingston, this project sought to integrate primary care with creative community engagement (Abel and Clark 2022). The initiative included both activities that required ambulation of participants and activities that occurred in people’s homes, mostly for those who are terminally ill and who cannot ambulate. Using a cohort retrospective analysis, researchers evaluated the effects of several interventions on visits to the emergency department from 2014–2017. First, people who were at risk for unplanned hospital visits were identified. The inclusion criteria were reportedly extremely non-specific:

“It was not limited to those with long-term conditions or older people, and anyone who gave cause for clinical concern could be referred into the service.”(Abel et al. 2018, p. e804)

Then, a care plan was drawn. It is reported that,

“Care planning was performed with a high degree of reliability,” (Abel et al. 2018, p. e804)

but without any explanation as to how this was assessed. Lastly, participants were linked with an employed ‘health connector’ charged with integrating them into a comprehensive, community-based service that included 5 components:

1. Web-based directory of available services in the community, such as housing.
2. Mapping of local social groups such as peer support groups and creation of new groups in case gaps were identified. One of the groups that was created, for instance, is a leg ulcer club.
3. One-on-one work together with an employed health connector to develop and enrich support networks.
4. Use of volunteered community connectors trained to facilitate use of the directory mentioned in 1 as well as local services.
5. Creation of specific support groups by the Health Connections Mendip^1^ organization that oversaw the project, for instance weekly Talking Cafes.

At the end of the study period, hospital admissions (which in the UK means visits) were reduced by 14% and associated costs were reduced by 20%. Costs of the complex intervention, however, are not reported, although the authors do state, without explaining, that,

“The return in investment for Frome was £6 for every pound spent.“(Abel et al. 2018, p. 809)

During the study period, Somerset saw an increase in hospital visits and associated costs.

From a methodological and public health perspective, the study has many flaws beyond those briefly mentioned above, i.e. no firm inclusion criteria, no inter-observer validity assessments, and no calculation of overall costs. First, the study is not controlled. Second, the intervention was overall complex, including several components that go well beyond standard SP-it was indeed quite a ‘system’. Third, no formal cost-effectiveness analysis was conducted. Fourth, other indices of success, such as personal well-being and loneliness, were not assessed. Fifth, since the study is cluster-based, the effects of the intervention on individual participants was not assessed.

In terms of level of evidence, robust randomized controlled studies (RCT) or systematic reviews are considered to be the gold standard. Since there is no robust RCT to measure the effects of SP on loneliness, we are left with systematic reviews.

A 2021 systematic review focusing mostly on the outcome of loneliness reduction reviewed 9 studies of SP, all done in the UK. The review stated that SP was seen favorably by service providers and participants, but nevertheless concluded that,

“the variability and paucity of evidence and lack of control group comparisons make it difficult to draw conclusions regarding the impact of the SP model on loneliness in particular, or on public health in general.”(Reinhardt et al. 2021, p. 211)

Similarly, a 2022 systematic review of SP in primary care that explicitly sought to focus on research outside of the UK (meaning that the research protocol did not have any language or country of origin restrictions) evaluated 68 reports from 53 different research projects. Fifty-one of these projects were conducted in the UK, and two in Australia. Fifty projects had no control group, and only two of these were randomized. The review concludes that,

“Uncontrolled studies with shorter time frames showed mostly positive effects, while effects reported from studies with control groups or longer follow-up periods were smaller or inconsistent.” https://doi-org.eproxy.lib.hku.hk/10.1001/jamanetworkopen.2022.36676

This means that the risk of bias is high in studies demonstrating reduction in loneliness.

Lastly, a 2022 meta-synthesis reviewed qualitative evaluations of SP interventions by participants in both English and French. The review included studies from the UK, Canada, the United States, and Sweden. Notably, out of the 19 publications reviewed, one was a government report and one a PhD thesis (Liebmann et al. 2022).

In these publications, participants reported that SP activities increased their sense of well-being, including increased sense of belonging to the community, improved self-confidence and self-worth, increased sense of purpose, pride and achievement, and distraction from personal issues. An example for improved confidence thanks to SP can be seen in the following quote:

“After [my partner] passed away I was, not a recluse, but I just didn’t want to talk to anybody. But since I’ve been coming to see [the link worker] I’ve broadened my horizons and I get out … I’ve got a lot more confidence” (Male, 60–64 years) (Liebmann et al. 2022)

At the same time, the meta-synthesis highlighted several reported reasons explaining the failure of SP for some participants: the connection with, and attitude towards the link worker, different interests compared to the other participants in the program, lack of interest in the activity associated with the program, and poor emotional state/lack of motivation to engage with link worker and other participants.

The authors themselves note that the effectiveness of SP interventions might be difficult to prove because it might depend on the nature of the activities themselves. (Liebmann et al. 2022) Further, social prescribing may not be helpful for those who are lonely without being socially isolated. They suggest that SP activities should be individually tailored, and that they be distributed equitably, paying attention to people living in deprived areas with little access to medical general practitioners who are supposed to initially socially prescribe. Furthermore, the authors note that SP might require continuous engagement rather than a limited pre-defined set of social encounters. In other words, SP may only be effective as long as it formally continues, and so management of loneliness in an individual would in such cases be akin to treating chronic diseases such as hypertension and diabetes rather than an acute condition.

Lastly, a non-randomized study of 114 adult community dwellers in Brisbane, Australia, that was not included in the reviews above, examined loneliness, well-being, and health service use in the past 2 months as primary outcomes, and social anxiety, psychological distress, and social trust as secondary outcomes in two groups. The control group received standard of care from the primary care physician, while the intervention group received standard of care plus social prescribing (the nature of which was not described). After 2 months, participants in the intervention group reported statistically significant decreased rates of loneliness only as measured directly (by explicitly asking about loneliness in the recent period) and increased rates of social trust; rates of loneliness as measured by an 8-item UCLA survey did not show a statistically significant variation (Dingle et al. 2024).

To summarize: while certainly plausible from a theoretical perspective, and arguably suggested by empirical literature, we do not really know whether SP reduces loneliness, depression, suicide rates, and healthcare usage, or increases wellbeing in the long run. It may, or it may not. The effectiveness of SP will ultimately depend on the lonely individuals themselves, their connection with the link worker, and the kind of activity involved. Furthermore, in order to mitigate loneliness, SP may have to be a continuous process rather than just culminating in several encounters. This would involve considerable resources, and would be challenging to evaluate based on cost-effectiveness indices. While these considerations and the paucity of evidence raise considerable justificatory difficulties for a public health policy grounded in justice and cost-effectiveness calculations, it may not be the case for a public health policy grounded in care ethics (Tomkins and Bristow 2023).

## Care ethics and public policy

This section first elaborates on the importance of care and highlights key characteristics of care ethics. It then distinguishes care ethics from a social justice approach, preparing the ground for Section "Loneliness and SP" where I argue that care ethics is more adept at explaining the intuition that SP policies are morally justified despite their current lack of empirical support and the public resources they require.

Care is fundamental to who we are- in fact, humans are what they care about (Frankfurt 1982). Care may be defined as a virtue, affect, or activity (Brake 2017; Bhandary 2020). The person who cares for others or has at least the disposition to care for others is usually considered to be a virtuous person. To care for someone is to wish someone well, for them to be well. Caring for someone may simply culminate in thinking of them, remembering them. But mental acts of care are often translated into physical acts of care. Caring for someone is to enact that wishing well, by preparing food, by clothing, by tucking in bed, by asking ‘how was your day’? In such instances, caring becomes caregiving. Caregiving can be material or emotional. Touch can be a form of caregiving. When you touch someone you offer them your body (in a very specific way), but you also offer emotional support. And when you touch, you are being touched back (Kearney 2021; Lederman 2026). We care, but we also want to be cared for and feel that we are being cared for. Being able to care and actually caring for others, and being cared for by others, are considered essential needs for many people and scholars (Brownlee 2020; Lederman 2024; Strauss 2024).

One of the central tenets of care ethics is that all humans require care at some point in their live (Baier 1995; Noddings 2002; Held 2006; Engster 2015; Greenswag 2019; Strauss 2024). Throughout life, and specifically in certain periods in life, we rely on others to sustain us. Not only that, but we also rely on a third party—society for instance—to sustain those who sustain us. Authors have normatively distinguished care during periods of dependency versus care during periods of independency (Brake 2017; Bhandary 2020) Such a distinction however, is unfortunate because it pulls the rug under the acknowledgment that all humans are vulnerable and depend on others all the time, perhaps with various moments in which we are especially dependent (Agich 1993; Have 2016; Greenswag 2019). Eva Kittay’s explicit strategy is to first account for the clearest cases of dependency, and then expand it to all humans qua dependent (Kittay 2001).

Care ethics goes beyond material or emotional dependency. Humans are inherently relational creatures in that we can only define and shape our identity as human beings, let alone acknowledge our identity, in a social environment that enables us to relate to other humans, specifically humans we want to relate to.^2^ Humans are also inherently relational creatures insofar as we often assess our wellbeing, our flourishing, based on the social relations we perceive to have. We are content if we believe that others respect and appreciate us; we become frustrated if we believe the opposite. In sum, humans depend on certain kinds and certain degrees of social relations throughout life. At some point in our lives, we become aware of such dependency.

The fact and realization of our universal dependency and inherent sociality motivate a *prima facie* moral duty to care. Such a duty is not a matter of coercion, but it is not voluntary either, in that we cannot simply choose who we wish to care for and about. It is analogous to familial duties: you do not choose the family you are born into or which you bring into the world, yet you have a *prima facie* duty of care towards it. This duty comes in degrees, perhaps as a function of the level of relatedness; it is not unlimited. In this way, we ought to care to some degree for all human beings qua fellow dependent, sociable creatures, and we ought to care to a higher degree for those who are closer to us (Engster 2015; Brownlee 2020).

At this point, two main difficulties arise. First, defining what such care actually means is of course necessary for a full articulation of this account. Such articulation, however, requires much more work (Slote 2007) Second, and relatedly, caring, particularly in the context of public health and policy, may sometimes require certain resources, and since resources are limited, there must be a way to distribute them. Accounts of justice as fairness, i.e. rather than utility (Rawls 1971), are particularly apt to address the latter difficulty, but how could a care ethics address them? This is in fact part of the challenge of figuring out,

“how to put responsibilities for caring at the center of their [societies] democratic political agendas.”(Tronto 2013, p. IX)

The move from social justice as it is traditionally understood within a liberal framework, particularly of rights and corresponding duties, to care ethics is about definitions and thresholds: what is ‘good,’ i.e. how is it defined and who defines it? what is ‘effective’? and what is harmful? It is about hermeneutics: who defines who the relevant stakeholders are and who interprets their needs and whether their needs are fulfilled (Greenswag 2019), From a justice perspective the focus on effectiveness and cost-effectiveness is high compared to care ethics, and so is the need to justify policies based on a cost-effectiveness analysis. Of note, perhaps the two most foundational texts in public health ethics emphasize effectiveness as a criterion to evaluate public health policies and infringement of individual rights (Kass 2001; Childress et al. 2002). My argument is not that policy makers should completely ignore considerations of cost-effectiveness. The argument is rather that these should be de-prioritized to make room for less quantifiable considerations as promulgated by care ethics. The move from justice to care ethics then is also about priorities:

“Few would hold that considerations of justice have no place at all in care. One would not be caring well for two children, for instance, if one showed a persistent favoritism toward one of them that could not be justified on the basis of some such factor as greater need. The issues are rather what constellation of values have priority and which predominate in the practices of the ethics of care and the ethics of justice.”(Held 2006, p. 15)

Care ethics is also highly contextual, operating in the realm of non-ideal theory (Engster 2015; Greenswag 2019). Understood as a focus on interpersonal relationship and decision-making based on contextual rather than principled consideration, care ethics has been found empirically to best explain parents’ medical decision making at the end of life of their children (Neefjes 2023). Specifically, in stark contrast to considerations of justice, it is concluded that in cases of young children with a short life expectancy due to illness parents try to ensure the best life for their children even “as the needs of other family members are not prioritized,” and that “…children with an LLC [life limiting condition] come first within their family.”(Neefjes 2023, p. 1738).

Resource allocation in the spirit of care ethics, then, starts with and is motivated by the identification of the concrete needs of particular individuals whose positive claims to these needs are judged to be compelling. Furthermore, resource allocation in the spirit of care ethics is and should be determined and assessed primarily by contextual, emotional etc. considerations, and only secondarily by justice considerations (Noddings 2002; Held 2006, p. 10). While some degree of scrutiny is inevitable in fulfilling the expressed or otherwise needs of the children, parents generally provide for them. There is little room for a principled allocation scheme to divide resources between the parents and them, or among the children themselves. Instead, parents (are expected to) just give and give, often sacrificing their own needs and interests for their children. Similarly, context and emotions often determine resource allocation among the children when those who scream the most or who evoke empathy receive more than their fair share. The end goal is not some measurable outcome, or at least not a measurable outcome that is justifiable by considerations of justice. Rather, parents give because they care (ideally); considerations of justice might be important, but are secondary (Baier 1995; Noddings 2002; Held 2006; Strauss 2024).

The analogy between family and community, of course, breaks down at some point. Parents’ moral (and legal) obligations towards their children are stronger than citizens’ obligations to their co-nationals. Similarly, a democratic liberal state that demands taxes from its citizens has a moral obligation to account for the way it allocates this tax-payer money and to invest it rationally. A public health system that allocates resources to certain patients just because they are the noisiest is not sustainable, let alone praiseworthy. Furthermore, public health interventions that have empirically been shown to be effective are more likely to lead to positive and sustainable health impacts (Crane et al. 2024).

And yet, many healthcare interventions are being implemented without conclusive evidence of their cost-effectiveness (Crump 2008). There may be many reasons for this ‘evidence-policy gap.’(Dam et al. 2023). Some of these reasons are ethically unjustified, but some are. First, many public health interventions are complex, involving different measures and implemented over many years. Proving their effectiveness, then, becomes challenging. Second, once there is some minimal physiological or prior empirical reason to suspect that such interventions may be effective, then there is reason to implement them along with rigorous and continuous monitoring. Third, and most important here, devising and implementing any public policy is primarily an ethical (and political) endeavor, determining which problems merit attention in the type of society we want to have (Greenswag 2019). Just think back to the 2013–2016 Ebola virus outbreak in West Africa, where unproven vaccines were used as part of ‘compassionate’ care. Despite being highly controversial, compassion and the value of trust in the healthcare system and transitional (rather than distributive) justice eventually outweighed the need to prove their cost-effectiveness. Authors, in fact, explicitly admitted that such compassionate use “would negate the principles of distributive and social justice.”(Folayan et al. 2014) This does not mean that every compassionate use of every therapeutical measure of any public policy is justified (Zettler 2015). But it does provide historical precedence, allowing that some cases may be justified.

The next section argues that SP interventions are ethically justified. It first asserts a human right against loneliness, relying on existing accounts. Second, it argues that in the realm of non-ideal theory and political judgment, care ethics may be more adept at justifying SP than a strict social justice approach to public health. SP policies and efforts to implement and sustain them on a national level are worthwhile because they represent caring for individuals who are lonely or who are at risk of being lonely. The distinction between ideal and non-ideal theory is important here. The argument is not that care ethics is better than justice in addressing loneliness in the realm of ideal theory. Rather, the argument is that, once a right against loneliness based on an ideal theory has been established, SP may be better justified using a care ethics approach rather than a justice approach to public health, in light of the evidence that is currently available. This argument, then, aims at defending the intuition that SP policies are a good thing that should be increasingly implemented and researched worldwide. If the evidence changes, the argument may change as well.

## Loneliness and SP

The immediate answer to the question ‘why should individuals and governments care about human loneliness’? is that humans have a right not to be lonely. They have a legitimate claim to the conditions necessary for them not to be lonely. The next question becomes ‘what foregrounds such a right?’ Authors have articulated and defended such a right (or a variation of it such as a right against social deprivation or a right to belong- the differences between these are irrelevant here), using various strategies.

One strategy is phenomenological, focusing on the inter-human encounter that is inherently essential for our being, and having an identity, in the world, therefore becoming normative (Stauffer 2015). Such a strategy, while theoretically powerful, remains moot on a practical level when it comes to societal obligations and questions of resource distribution. A theory that merely substantiates the idea that our interactions with other persons are phenomenologically valuable tells us little about the kinds of material or nonmaterial resources we deserve. Resorting to an alternative or additional strategy then, becomes in any case necessary.

Having access to a certain quantity and quality of social relations can be justified based on human dignity (Brownlee 2020). It can also be perceived as necessary for wellbeing (Powers and Faden 2008; Brownlee 2020) or for justice as capabilities (Nussbaum 2007), and thus derive from the right to wellbeing or to justice, respectively. Framed as a social determinant of health, a right to access to appropriate social relations can also be justified as derivative of the right to health. (Lederman 2023a, b, c).

Below I critically review two modified Rawlsian-justice approaches to justify a right to a specific kind of social relations- caring relations. A sufficientarian, or egalitarian social justice strategy holds that members of a given society prima facie deserve X to a minimally sufficient degree, or to a degree that is equal or equitable to their peers. While ‘X’ may be many kinds of goods, e.g. opportunities, welfare, capabilities, resources etc. in the case of loneliness it may be understood as opportunities to affiliate or interact with other humans; actual (rather than opportunities for) interactions with others; (Brownlee 2020) health or access to healthcare (Lederman 2023a, b, c). In the case of sexual or erotic loneliness X may mean a prima facie entitlement to governmental and institutional interventions that facilitate access or opportunity to a sufficient quantity and quality of intimate relationship, touch, companionship, physiological fulfillment etc (Lederman 2026).

Loneliness, however, is an affective, subjective state- how can we reasonably account for peoples’ experiences of loneliness and allocate resources to support them? Doing so within a democratic, liberal framework seems particularly challenging. Individuals will vary in the kind and quantity of the conditions they need in order not to be lonely, and determining impartial thresholds above which individuals should not be lonely would prove difficult. The right against loneliness may then encounter the same issues plaguing the right to health- if the corresponding duties turn out to be overly demanding, then the more likely it is that the relevant right is implausible (O’Neill 2005). Bracketing these difficulties for the moment, this essay asserts that individuals in a community that is committed to optimizing their wellbeing and flourishing do have a right against loneliness. This right corresponds to governmental duties to a variable degree, depending on how perfectionist (Raz 1986) or communitarian (MacIntyre 2007) the community is. The main argument advanced in this paper is that such duties include financing SP interventions. How so?

### Caring for the lonely means financing SP interventions

Addressing the needs of lonely individuals within a liberal democratic regime may begin by examining a similar attempt by care ethicists to accommodate obligations and conditions for caring in general within such a regime (Greenswag 2019). Joan Tronto, for example, argues that current democracies should be re-configured to move away from market-based, economic considerations in public policy towards greater integration of caring practices, where citizens care with other citizens for their democracy and in turn receive care from their democratic leaders (Tronto 2013). However, a case to augment care practices may also be grounded in social justice, specifically in social contract accounts of liberal regimes (Kittay 1999; Bhandary 2020). In other words, for the sake of argument, while this paper explicitly distinguishes a social justice approach from a care ethics approach, the two can and indeed have been reconciled.

In one of the most developed justice-based account to strengthen care practices, Elizabeth Brake argues that caring relations should be added to the Rawlsian list of primary goods to ensure equal access to the social and legal bases of caring relationships (Brake 2017). Caring relationships mean relationships that are intimate, ongoing and reciprocal:

“Citizens, then, have a claim to equal access to the social and legal bases of caring relationships and a claim that the state arrange institutions in a way which promotes the formation of such relationships.”(Brake 2022, p. 213)

Brake introduces her argument to accommodate for human dependency within a justice framework, responding to those challenging such commensurability. But two main counter-arguments could be produced, one against the reliance on Rawls generally and one specific to the discussion here, basically challenging the relevance of her argument to loneliness as a concrete public health problem.

First, Brake admits being a political liberal, which means that law and policy could not according to her be based on substantive moral claims. Indeed, she is committed to a thin theory of the good (Brake 2017). The problem for Brake, however, is that different people would want and need variable quantities and qualities of care relation as a primary good. For one, making and having food on the table at the end of the day is an essential part of caring relations, while for another it is having someone to Zoom with before heading to bed. For others still, caring and being cared for may mean working extra hours to allow for the partner to purchase the most expensive shoes in the market. From a public policy perspective, since resources are limited, justice will require us to decide which forms of care are worthy and which are not. In other words, we will need to decide what form of care is good or at least good enough to publicly support. Making a claim that humans deserve as part of justice to be part of a specific kind of relations—what she defines as a caring one—thus constitutes a thick normative claim. She may be criticized then for introducing a perfectionist normative account of interpersonal relations into a political liberal paradigm, which is incommensurable (Arneson 1990). Relatedly, asserting a justice claim to a fair opportunity to care relations is not the same as asserting a justice claim to a certain quality and quantity of care relations. Brake in fact admits this when she allows that not all people who demand certain relations should indeed receive them, based on individual characteristics, e.g. being obnoxious, or contextual conditions, e.g. having overly burdened unpaid care providers. As Richard Arneson puts it,

“To the extent that we agree that from the standpoint of distributive justice it is best to measure people’s resource holdings by a primary goods standard, our distributive principles should be sensitive to people’s opportunities for primary goods, not their actual lifelong primary goods shares, despite Rawls’s commitment to the latter.”(Arneson 1990, p. 444)

Relying on empirical data, Brake responds that since a caring relationship is essential for mental health this should be the minimal threshold:

“The relationships that provide widespread, non-fungible mental health benefits are stable relationships with the same people, involving mutual concern for one another’s welfare and frequent interaction. This would rule out a minimal conception of care as simply exchanging money or performing services, and it would rule in close, life structuring friendships and small-group polyamory… It would, notably, rule out care by robots.”(Brake 2017, p. 138)

Such a response, however, raises more difficulties. The first difficulty is that mental health or health in general is also not included in Rawls’ primary goods and including it requires an argument (Daniels 2007; Filk 2007). A second difficulty is that, even if we are convinced that the empirical evidence is sufficient to show that a caring relation is the *only kind of* relation that can ensure mental health (which is not the case), some people may prioritize mitigating their loneliness over other aspects of their mental health, even if it means an abusive relation (or a relation with a robot) that in one sense jeopardizes this very mental health. Meaning, even if a certain relationship makes them depressed, they might still prioritize their being not lonely. Determining for them what kind of relation is preferable or what aspect of mental health is the most important one does not align with political liberalism.

A case in point is her quick dismissal of a caring relation with social robots, due to deception (Brake 2022). This argument was shown to be weak (Blackford 2012) and, more significantly, seems to introduce normative content that no committed liberal could ever accept. Besides, some users are fond of their social robots, and social robots may at least be a temporary or last-resort measure against loneliness and social isolation for some individuals (Lederman and Jecker 2023; Saslow 2026).

The second counter-argument to Brake is that Rawls’ primary goods apply to an ideal local context, or polity. Rawls’ primary goods apply solely to basic institutions under the normal functioning of a polity. The assumption here, however, is that liberal democracies worldwide are facing a pandemic of loneliness, specifically affecting roughly 16% of people worldwide (Commission on Social Connection 2025). (omitted for review) (the claim here is not that rates of loneliness are higher than before, which is probably not the case) (Luhmann et al. 2023). So even if Brake’s suggestion is appealing under ‘normal’ conditions (which it certainly is, for a communitarian or a perfectionist liberal), it hardly applies here.

Brake herself admits her call from justice to galvanize care relationships is questionable in light of concrete considerations:

“resources devoted to such a program could be better used elsewhere–building homes for the homeless, tutoring underprivileged children, disaster relief, or overseas aid through the Peace Corps. As a question of resource allocation, this requires hard figures from specific jurisdictions.”(Brake 2017, p. 145)

In her self-critique, Brake is spot on. Championing more care and caring relations in public policies is intuitively compelling. As part of such caring, championing public policies to mitigate loneliness is intuitively compelling as well. At the same time, however, loneliness is an affective state- how can public reason possibly accommodate individuals’ feelings? Further, imagine that a certain policy cannot be justified based on concrete considerations, e.g. of cost-effectiveness? Should we simply abandon it? Highlighting the distinction between ideal and non-ideal theory may help explain Brake’s difficulty here.

### Ideal vs. non-ideal theory

An ideal theory has been usefully compared metaphorically to a map: it is a truth tracker rather than truth maker; it gives you a general direction but at the same time it is always outdated. It cannot possibly inform you about current traffic, and concrete, real-world obstacles (Schmidtz 2011). Rawls himself refers to his ideal theory only as the first step in devising real-life public policies. The next step is political judgment which is very much attuned to concrete empirical facts and sentiments (Rawls 1971; Engster 2015).

Making an ideal theory case for the right of individuals not to be lonely is one thing. And imagining such an ideal utopia is indeed worthwhile and helpful (Stahl 2023). Detailing what this involves from the standpoint of governments/ societies and individuals is quite another. As mentioned, the assumption here is that the theoretical basis for a right not to be lonely is sound. Ideally speaking, this means that societies must ensure that such a right is respected. The devil, however, is in the details, and details are the business of non-ideal theory and political judgment (Engster 2015; Greenswag 2019).

Engaging in non-ideal theory, social justice approaches have increasingly been used to support and scrutinize public health policy (Gostin and Powers 2006; Powers and Faden 2008; Segal 2010; Hassoun 2020). Distributive, rather than restorative, justice arguments pertaining to public policy ought to consider expected consequences, or benefits, against the associated costs. Since resources are limited, formal cost-effectiveness analyses become necessary for evaluating policy. Making an argument from justice then to fund national campaigns for SP against loneliness would require an analysis showing the success of the various interventions of SP plotted against their costs. In the case of SP against loneliness, however, social justice justifications may be somewhat challenging, as the actual demonstrated value in terms of effectiveness of these interventions is limited.

The argument here is that rationality in the case of SP does not necessarily translate to or prioritize cost-effectiveness analyses that are often expected within a justice framework, or within economic-driven perceptions of politics. Rather, SP should be supported in a manner that de-prioritizes justice considerations of cost-effectiveness analyses because it expresses care for the lonely, and caring for the lonely is a good thing in and of itself. Why is caring for the lonely a good thing? Because individuals have a right against loneliness- they are entitled not to be lonely. And why should society care about the lonely while de-emphasizing the economic aspect? Because care ethics tells us so: it tells us that a political society should care for its members in this fashion, and that:

“we should expect from the state a certain kind of support for all of our caring practices.”(Tronto 2013, p. XI)

In the spirit of care ethics, the architects of the Compassionate Frome Project acknowledge our inevitable dependency:

“Sooner or later, however orderly our lives and no matter how much care we take to keep them secure, for most if not all of us some unanticipated event will shake the very core of our being.”(Abel and Clark 2022 p. 2)

Grounding demands of care on social contract argument seems only valid if they entertain a modified version of Kittay’s ‘dependency critique’: A conception of society viewed as an association of equals masks equitable dependencies behind a veil of ignorance that manifests as unequal dependencies and disadvantages in various temporal and contextual instances throughout life:

“While we are dependent, we are not well positioned to enter a competition for the goods of social cooperation on equal terms.”(Kittay 1999, p. xi)

Kittay’s argument is compelling. When it comes to loneliness mitigation, however, even this modified social contract account might not address the main objections mentioned above (which, of course, was not Kittay’s aim, so this is not a critique of her specific account). The first objection is that any account of the basic structure of a society, either ideal or non-ideal, could hardly apply to an extreme event, such as a loneliness pandemic. In this context, a social contract approach might not at all be relevant. The second objection is that public policies framed within a social or distributive justice framework usually require rigorous cost-effectiveness analyses. While neither of these difficulties is detrimental, one may evade them altogether or de-prioritize them by complementing or substituting a (an ideal or non-ideal) justice framework with a care ethics framework.

### Care ethics and loneliness

The normative strength of non-ideal care ethics lies exactly in the grounding of a right to be cared for—with a corresponding duty to care for— in our shared vulnerability and dependency; our shared and necessary need at least at some point in time for social interactions that define and nourish us. From this shared vulnerability and need for others, as well as from moral sentiments such as empathy and compassion, a shared duty to care for our fellow humans is born. (Agich 1993; Baier 1995; Noddings 2002; Held 2006).^3^

Care ethics has been espoused in discussions of policing in the UK, where authors have rightly prioritized need over principles or effectiveness:

“A care ethical approach to evaluation focuses on how an action or intervention will affect particular people in particular circumstances ahead of the abstract criterion of whether it is right or wrong or the instrumental criterion of whether ‘it works’.“(Tomkins and Bristow 2023, p. 122)

Public policies, then, should primarily be evaluated based on the general positive subjective and objective effect they have on individuals and on their function as symbolizing and demonstrating care, and only secondarily on indices of effectiveness. Or, put another way, the very act of defining and measuring effectiveness, as well as deciding who should be the relevant stakeholders to assess effectiveness depends on the underlying political philosophy, and would vary greatly within a care ethics approach compared to justice (Engster 2015; Greenswag 2019). Within a care ethics approach, public policies should be evaluated based on whether they aim to express care, whether they indeed express care, whether they are perceived by relevant stakeholders to express care, and how they advance communities towards being communities in which care is a central value.

Loneliness is bad because it goes against the very fact of human inherent sociality: it potentially hinders our ability to develop, shape and express our own identity, and it means that we lack others who can sustain us, perhaps because they lack the resources to sustain us (MacIntyre 2007; Brownlee 2020; Lederman 2023a, b, c). At the very least then, care may minimally be defined as attempts by society as well as other individuals to prevent and mitigate loneliness. And limited resources then should be distributed so as to assure a certain level of care that is sufficient to mitigate loneliness as experienced by individual community members.

But here we encounter the same objection mentioned above- What is a sufficient level? Sufficient to whom? What should be the loneliness-related outcome? reduction in prevalence of all or some specific types of loneliness or rather the kind of loneliness that is most likely to cause adverse health effects? Perhaps most importantly, what kinds of care practices would be justified here?^4^ Caring may involve practices that are intuitively objectionable, such as the provision of cigarettes or alcohol, or in the case of sexual loneliness of incels, facilitated access to women (Räsänen 2023, 2024). These practices may still express care, but at the same time there is something about them that makes them ethically wrong. Moreover, if we care about someone we also do not want to waste their time, and if SP does not really work, then it will just be a waste of time for everyone involved.

I have argued that in light of the limited proven effectiveness, justice-based theories would be hard-pressed to answer these questions. Within care ethics, two complementary answers can be given.

First, care ethics de-prioritizes outcome and prioritizes need. The need is to perceive to be provided with care and indeed to receive care; it is the need to know that society and individuals in society are doing something to help you feel less lonely, rather than actually reducing your loneliness. Policies and resource allocation grounded in care ethics are motivated primarily by moral sentiments such as compassion and empathy, and only secondarily by cost-effectiveness analyses. The ‘Compassionate Frome Project’ is thus praiseworthy for its espousal of compassion as the bedrock for public health policy.

Compassion towards the lonely means national mobilization of resources to invest in interventions even without a proper cost-effectiveness analysis or in cases where such analyses are inconclusive. Cost-effectiveness is not the main index used to evaluate policies and interventions within care ethics. Rather, these are primarily evaluated based on several other indices: to what extent they do express compassion and care and whether they are indeed perceived by the lonely to express care; whether they are ethically worthy in and of themselves and whether they are the kind of policies and interventions we would like to have in a given community etc. In other words, policies and interventions should be assessed based on whether they indeed reflect a care ethics approach that acknowledges our shared vulnerabilities and dependencies and whether they express the kind of care considered to be praiseworthy. The latter will be judged based on the concrete political philosophy that we espouse and our general conception of the good.

Another consideration is of course the kind of risks they impose on individuals and their associated costs to the community: the lower the risks and costs, the more likely they are to be justified. Assessments would be political and ethical, conducted by community members, ethicists, and policymakers.

SP interventions, meant to connect individuals back to the communities, are justified *prima facie* merely because they symbolize and express care in the way we would like human communities to express care. They assume that humans are lonely because they lack the kind and quantity of needed social relations with other humans and they aim to facilitate fulfilling that need. The social relations SP interventions aim to facilitate are generally considered to be a good thing (unless they are abusive etc.). There is reason to assume they are effective, at least as long as they are being implemented, and they have the potential to provide a long-term solution to loneliness that goes beyond the intervention itself- meaning they can lead to steady, long term, amicable or even romantic relationships. Even if SP interventions are not effective, they carry minimal risks for individuals (in terms of costs, different interventions vary greatly).

The second answer to the concerns raised above regarding sufficiency and desired outcome is urgency. Caring for the lonely in the midst of a loneliness pandemic means mobilizing resources *urgently* (omitted for review). Millions of people are currently suffering and dying from loneliness in various countries across the world (Commission on Social Connection 2025). More research is indeed needed (Husk et al. 2019), but lack of proof should not hinder care that is needed *now*. The lonely may not bleed as those dying of Ebola or grasp for oxygen as loudly as those dying of Covid-19, but their plea to be cared for is just as visible and loud.

## Conclusion

The ethics of loneliness must face at least two major challenges. One is to theoretically establish a right against loneliness. The second is to articulate what such right entails exactly. This paper addresses the second challenge, heeding in fact Joan Tronto’s call to specify what care ethics would mean in real life. The paper specifically discusses what care ethics would entail for public policy concerning loneliness mitigation, focusing on social prescribing. The paper first reviews available empirical evidence regarding SP, one of the most common and promising interventions against loneliness. The evidence, unfortunately, is not as strong as one would hope, and one of the conclusions is that rather than SP in general researchers may be wiser to measure the effects of particular interventions, i.e. community classes, community cafes, etc. The paper then distinguishes ideal from non-ideal theory and state of affairs, practically dividing the two major challenges above into different realms of inquiry. It argues that in light of the paucity of empirical evidence, and the resulting difficulty in justifying such interventions within a distributive justice and social justice framework that prioritizes cost-effectiveness analyses, SP should be justified by espousing a care ethics approach that prioritizes specific needs in specific circumstances while de-prioritizing cost-effectiveness analyses.

## Footnotes

1 Home—Health Connections Mendip accessed 4/2024.

2 Opponents may argue at this point that humans can also develop and sustain their identity through relations with AI. This issue will be addressed elsewhere.

3 Such a duty could readily be expanded to non- human entities such as animals and ecosystems, but this discussion is beyond the scope of this paper.

4 I thank an anonymous reviewer for raising this insightful point.
